# Synthesis, Cytotoxic Activity, and DNA Binding Properties of Copper (II) Complexes with Hesperetin, Naringenin, and Apigenin

**DOI:** 10.1155/2009/347872

**Published:** 2009-10-08

**Authors:** Mingxiong Tan, Jinchan Zhu, Yingming Pan, Zhenfeng Chen, Hong Liang, Huagang Liu, Hengshan Wang

**Affiliations:** ^1^The Key Laboratory for the Chemistry and Molecular Engineering of Medicinal Resources, School of Chemistry & Chemical Engineering, Guangxi Normal University, Guilin 541004, China; ^2^Department of Chemistry and Biology, Yulin Normal College, Yulin 537000, China; ^3^Department of Pharmacology, Guangxi Medical University, Nanning 530021, China

## Abstract

Complexes of copper (II) with hesperetin, naringenin, and apigenin of general composition [CuL_2_(H_2_O)_2_] ⋅ nH_2_O (**1**–**3**) have been synthesized and characterized by elemental analysis, UV-Vis, FT-IR, ESI-MS, and TG-DTG thermal analysis. The free ligands and the metal complexes have been tested in vitro against human cancer cell lines hepatocellular carcinoma (HepG-2), gastric carcinomas (SGC-7901), and cervical carcinoma (HeLa). Complexes **1** and **3** were found to exhibit growth inhibition of SGC-7901 and HepG2 cell lines with respect to the free ligands; the inhibitory rate of complex **1** is 43.2% and 43.8%, while complex 3 is 46% and 36%, respectively. The interactions of complex **1** and its ligand Hsp with calf thymus DNA were investigated by UV-Vis, fluorescence, and CD spectra. Both complex **1** and Hsp were found to bind DNA in intercalation modes, and the binding affinity of complex **1** was stronger than that of free ligand.

## 1. Introduction

The success of cisplatin and its derivatives as anticancer agents has stimulated the development of metal-based compounds [1–4]. Recently, interests possessed in copper(II) complexes are increasing due to their possible medical uses as antitumor agents. And, new bioactive ligands, involving natural product ligands [5–7], have been applied for the design of Cu-coordination novel drugs; for that, naturally occurring compounds have served as a major source of drugs for centuries [8–10]. 

Flavonoids are phenolic compounds widely distributed in plants, which display a variety of biological activities, such as antioxidant, anti-inflammatory, blood lipid-lowing, and anticarcinogenic activities [11–13]. Many flavonoids are natural chelators and, flavonoid metal complexes have showed significantly higher cytotoxic activity than those of the parent flavonoids, such as quercetin, morin, and chrysin and so forth [14–16]. Besides, it is demonstrated that the coordination of copper(II) ion with bioactive ligands can actually improve the pharmaceutical activity of the drugs themselves and reduce their toxicity effects [17–19].

**Scheme 1:**
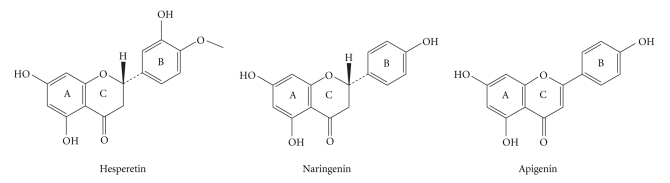
Structures of the ligands.

Hesperetin (5, 7, 3′-trihydroxy-4′-methoxy-flavanone, Hsp, Scheme 1), naringenin (4′, 5, 7-trihydroxyflavanone, Nrg, Scheme 1) and Apigenin (4′, 5, 7-trihydroxyflavone, Apg, Scheme 1), are biologically active flavonoids, commonly found in fruits and vegetables [20]. They have been reported to exhibit antitumor effects against breast cancer and hepatoma HepG2 cell lines [21, 22]. In addition, some metal complexes of Hsp and Nrg have been found to exhibit antioxidant and anticancer activities [23, 24]. Nrg schiff-base La(III) complex against the A-549 cell line was more potent than cisplatin at most experimental concentrations [25, 26]. Also, Cu(II) complex of Nrg schiff-base possessed potent antioxidant activity and better than standard antioxidants like vitamin C and mannitol [27]. 

The aim of this study is to prepare new copper(II)-based antitumor compounds and investigate the synergistic effects upon flavonoids coordination with Cu(II) ion. In this paper, three copper(II) complexes with Hsp, Nrg, and Apg have been synthesized and characterized by elemental analysis, UV-Vis, FT-IR, ESI-MS, and TG-DTG thermal analysis, and their cytotoxic activities were estimated against HepG-2, SGC-7901, and HeLa cell lines in vitro. The binding properties with calf thymus DNA of Hsp and its Cu(II) complex, which have good activities against the tested cell lines, were investigated through UV-Vis, fluorescence, and circular dichroism (CD) spectroscopy.

## 2. Experimental

### 2.1. Instrumentation and Materials

Hesperetin, naringenin, and apigenin were purchased from Shaanxi Huike Botanical Development Co., Ltd., and the products (compounds purity > 98%) were identified by spectral methods. The solvents and metallic salts used were analytical grade. All the materials were used as received without further purification unless noted specifically. Tris-HCl-NaCl buffer solution (5 mM Tris, 50 mM NaCl, pH was digital adjusted to 7.35 by titration with hydrochloric acid using a sartorius professional meter professional meter, tris(hydroxymethyl)aminomethane) (Tris) was prepared using double distilled water. Calf thymus DNA (ct-DNA) was purchased from Sino-American Biotech. Co. Ltd, Beijing. A Tris-buffer solution of ct-DNA gave a ratio of UV absorbance at 260 nm and 280 nm of *ca. *1.8 ~ 1.9 : 1, indicating that the DNA was sufficiently free of protein. The DNA concentration was determined spectrophotometrically from the molar absorption coefficient (6600 M^−1^cm^−1^) at 260 nm. Stock solutions were stored at 4°C and used no more than 4 days after preparation. Infrared spectra were obtained on a Perkin-Elmer FTIR Spectrometer. Elemental analyse was carried out on a Perkin Elmer 2400 Series II CHNS/O elemental analyzer. TG-DTG thermal analyses of the complexes were investigated on a thermogravimetry differential thermal analysis apparatus (Pris Diamond, America). UV-Vis absorption was performed on a Varian Cary100 UV-Vis spectrophotometer. Fluorescence measurements were performed on a Shimadzu RF-530/PC spectrofluorophotometer. The Circular Dichroic spectra of DNA were obtained by using a JASCO J-810 automatic recording spectropolarimeter operating at 25°C. The region between 220 and 320 nm was scanned for each sample.

### 2.2. Synthesis of Complexes

Synthesis of [Cu(Hsp)_2_(H_2_O)_2_] ⋅ H_2_O (**1**): An ethanol solution (10 mL) of Hsp (0.0604 g, 0.2 mmol) was added to a aqueous solution (15 mL) of CuCl_2_ ⋅ 2H_2_O (0.0204 g, 0.12 mmol) and was adjusted pH to 7-8 with ammonia solution. The mixture was refluxed with stirring for 12 hours, and brown precipitate formed during reflux and then allowed to cool to room temperature and filtered. The solid was washed with water and ethanol, and then air-dried for 2 days. Calc. for [Cu(Hsp)_2_(H_2_O)_2_] ⋅ H_2_O, C, 53.37 ; H, 4.48. Found: C, 53.68; H, 4.51%. IR (KBr): 3423, 1614, 1564, 1375, 1166 cm^−1^. ESI-MS: *m*/*z* 703. 

Synthesis of [Cu(Nrg)_2 _(H_2_O)_2_] ⋅ H_2_O (**2**): this green compound was synthesized by the same method as that employed for** 1.** Calc. for [Cu(Nrg)_2_(H_2_O)_2_] ⋅ H_2_O, C, 54.59; H, 4.28. Found: C, 54.75; H, 4.26%. IR (KBr): 3401, 1613, 1566, 1374, 1254 cm^−1^. ESI-MS: *m*/*z* 642.4.

Synthesis of [Cu (Apg)_2 _(H_2_O)_2_] (**3**): this yellow-green compound was synthesized by the same method as that employed for** 1. **Calc. for [Cu (Apg)_2_(H_2_O)_2_], C, 56.47 ; H, 3.48. Found: C, 56.81; H, 3.76%. IR (KBr): 3413, 1626, 1597, 1356, 1176 cm^−1^. ESI-MS: *m*/*z* 638.

### 2.3. Cytotoxic Activity Assay In Vitro

Cell lines: human cancer cell lines hepatocellular carcinoma (HepG-2), gastric carcinomas (SGC-7901), and cervical carcinoma (HeLa) were obtained from Shanghai Cell Bank in Chinese Academy of Sciences. Cell lines (Gibco, Scotland, UK) were grown in Dulbecco's modified Eagles medium (DMEN) at 37°C in a humidified atmosphere of 5% CO_2_/95% air. Cytotoxicity evaluation: assays of cytotoxicity were conducted in 96-well, flat-bottomed microtitre plates. The supplemented culture medium with cell limes was added to the wells. Compounds were dissolved in the culture medium with 1% DMSO to various concentrations, and the solutions were subsequently added to a set of wells. Control wells contained supplemented media with 1% DMSO. The microtiter plates were incubated at 37°C in a humidified atmosphere of 5% CO_2_/95% air for a further 3 days. Assessment of cytotoxicity was carried out by using a modified method of the Mosmann-based 3-(4, 5-dimethylthiazol-2-yl)-2, 5-diphenyltetrazolium bromide (MTT) assay. At the end of each incubation period, MTT solution (10 *μ*L, 5 mg/mL) was added into each well, and the cultures were incubated further for 4 hours at 37°C in a humidified atmosphere of 5% CO2/95% air. After removal of the supernatant, DMSO (150 *μ*L) was added to dissolve the formazan crystals. The absorbance was read by enzyme labelling instrument with 570/630 nm double wavelength measurement.

### 2.4. DNA Binding

The UV-Vis absorption titrations, fluorescence emission spectra, and CD absorption spectra studies were performed at room temperature. Hsp and its Cu(II) complex were all dissolved in a small amount of DMSO due to their poor solubility, then diluted to a concentration of 2.0 × 10^−5^ mol/L with Tris-HCl-NaCl buffer solution (5 mM Tris, 50 mM NaCl, pH = 7.35). Absorption titrations were performed by using a fixed compound concentration (2.0 × 10^−5^ mol/L); ct-DNA stock solution (1.0 × 10^−4^ mol/L) was added 30 *μ*L each time and gradually increased up to a sufficient concentration for studying. While measuring the absorption spectra, the solutions were allowed to incubate for 10 minutes before the absorption spectra were recorded, and an equal amount of ct-DNA was added to both the compound solution for the reference solution to eliminate the absorbance of ct-DNA itself. Fluorescence Emission spectra of compounds were performed by using a fixed compound concentration (2.0 × 10^−5^ mol/L) and an increasing amount of DNA (1.0 × 10^−4^ mol/L). Samples were observed between 200 and 800 nm. The CD absorption spectra of ct-DNA (1.0 × 10^−4^ mol/L) were recorded in the absence and presence of compounds (2.0 × 10^−5^ mol/L). The sample solutions were mixed and allowed to equilibrate at room temperature for 60 minutes before measurements. Each sample solution was scanned in the range 200–400 nm UV-Vis region with a screening rate of 100 nm/min at room temperature.

## 3. Results and Discussion

### 3.1. Synthesis of the Complexes

The Cu(II) complexes were synthesized by aqueous solution of CuCl2 · 2H2O and the ethanol aqueous solution of Hsp, Nrg or Apg in 1 : 2 molar ratio (metal salt/ligands). Ligands were deprotonated by adding ammonia solution. The structures of copper(II) complexes were characterized by elemental analysis, UV-Vis, FT-IR, UV–Vis, TG-DTG, and ESI-MS measurements. 

The elemental analysis data of the copper(II) complexes agreed with the theoretical values within the limit of experimental error. The ESI-MS of complexes were investigated in H_2_O-DMSO (1 : 1) solution, the characteristic peaks at *m*/*z* 703 and 642 corresponding to [M+H-H_2_O]^+^ of complexes **1** and **2**, and that of peak at *m*/*z* 638 assigning to [M +H]^+^ of complex **3**. 

UV-Vis spectrum of free ligands and their Cu complexes suggested that 4-keto and 5-hydroxy region of the flavonoids may be considered as the possible chelating sites. For example, free Apg exhibits an UV-Vis absorption maximum in DMSO solution at 206–250 nm, corresponding to the A ring portion, and a week band at 268–316 nm, corresponding to the B ring portion. Upon binding with the Cu(II) ion, forming complex **3**, the maximum absorption band is shifted to 235–286 nm and the weak band shifted to 290–340 nm with respect to the free flavonoid, suggesting an interaction of the Cu(II) ion with the condensed ring of the flavanone in positions 4 and 5. These results are in agreement with the results of others, [28–31] which indicate that this band shift is caused by the binding of the metal ion in this position. 

 FT-IR spectrum data in KBr of free ligands and their Cu complexes were compared in Table 1. The absorption around 3200 cm^−1^ due to phenolic hydroxyl in the free ligands shows significantly spectral change in all the Cu complexes, indicating the chelate formation through hydroxyl group. The Cu complexes show a medium broad band around 3400 cm^−1 ^ indicating coordinated water, later confirmed by TG-DTG thermal analysis. The intense absorption bands due to *ν* (C=O) of free ligands at 1630–1650 cm^−1^ has also shifted to lower frequencies at 1623–1626 cm^−1 ^in the Cu complexes. Thus it suggests that flavonoids are coordinated with Cu(II) through oxygen atoms of –OH and C=O groups [31]. This contention is further confirmed by the presence of *ν* (M–O) bands at about 600 cm^−1^ in the far IR frequency region. 

**Figure 1:**
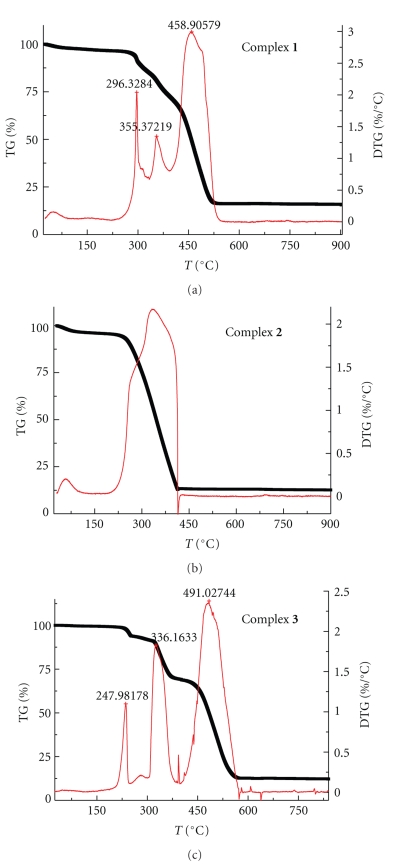
TG-DTG curves of the Cu(II) complexes.

The TG-DTG curves and thermal data for the dehydration and decomposition of the complexes are given in Table 1 and Figure 1. The results showed that the thermal decomposition of complex **1** displayed three stages from 30°C to 600°C. The first stage of decomposition from 30°C to 100°C was connected with the dehydration processes. The mass loss value of 2.09% was involved in the loss for two water molecules in the outer coordination sphere, which was in close agreement with the calculated mass loss value of 2.6%. The next dehydration processes appeared in the temperature ranges from 250°C to 300°C. In fact, it was very difficult to distinguish between the dehydration steps for inner and outer spheres from the graphic data. However, the total observed mass loss value of 5.2% was in close agreement with the calculated mass loss value of 5.13% for the two coordinated water molecules. The last mass loss stages were considered as the case of the decomposition of the ring breaking of the ligand and the formation of metal oxide. Complex **2** exhibits a sharp weight loss at temperature of about 253°C–440°C, which corresponds to the degradation of the ligand molecules and the formation of metal oxides. The dehydration process occurs at temperature range of 30°C–94°C and 124°C–253°C, corresponding to the outer sphere and coordinated water molecules. The thermal decomposition process of complex **3 **is basically the same as that of complex **1**. The dehydration process occurs in only one mass-loss step up to 247°C with 5.2%, which is in close agreement with the calculated mass loss value of 5.5% for the two coordinated water molecules in inner spheres. 

**Scheme 2:**
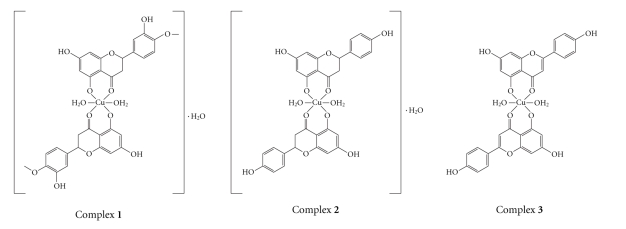
The possible structures of the Cu(II) complexes.

Based on these results, the possible structures of the complexes were suggested. As shown in Scheme 2, the copper(II) complexes have the general formula [Cu(L)_2_(H_2_O)_2_] ⋅ nH_2_O, where L is the ligands, and *n* = 0 for Apg, and *n* = 1 for Hsp or Nrg. The complexes are air stable for extended periods and soluble in DMF, dimethyl sulphoxide (DMSO), slightly soluble in Me_2_CO, and CH_3_OH, but scarcely soluble in water.

### 3.2. Cytotoxic Activity Assay In Vitro

**Figure 2:**
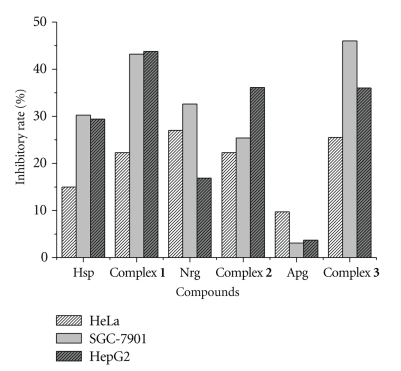
Inhibitory rate of complexes against the tested cell lines.

Cytotoxicities in vitro of Hsp, Nrg, and Apg and their copper(II) complexes have been estimated by MTT assay [32] against three typical human tumour cell lines involving HepG-2, SGC-7901 and HeLa. As shown in Figure 2, it is remarkable that complexes **1 **and **3 **exhibited cytotoxicity higher than that of complex **2 **against the selected cell lines. Against the SGC-7901 tumour cell line, the activity of complex **1** is 43.2%, while **3** is 46%, fifteen times stronger than that of Apg, and against HepG2, the activity of complex **1** is 43.8%, while complex 3 is 36%, ten times stronger than that of Apg. However, complex **2 **showed relatively significant inhibitory rate only against HepG2 cell line than that of Nrg.

Nrg and Apg are structurally similar with the same A and B rings, and Apg is a planar molecule in its rings A and C. The ligand Apg was found to show lower activities against the tested cell lines than the analogous ligand Nrg. However, the Cu(II) complex of Apg exhibited higher activity than the Cu(II) complex of Nrg, presumably the flavone planar structure of Apg is important for retaining its original planarity in the Cu(II) complex and plays an important role in the enhanced antitumour activity of the complex [33]. It is noteworthy that, Hsp and Nrg share similar structures but have different hydroxyl (OH) substitutions in ring B. In contrast to the inhibitory activity of the Cu(II) complexes of Nrg and Hsp, the high activity of Cu(II) complex with Hsp may be related to the different hydroxyl (OH) substitutions in ring C and the synergistic action of copper(II) ion with ligands [34–36].

### 3.3. DNA Binding Studies of 1 and Hsp

DNA is the primary pharmacological target of many antitumor compounds. Conformational changes of DNA directly affect genetic expression, which is closely related to carcinogenesis and anticarcinogenesis. Similarly, interactions between small molecules and DNA rank among the primary action mechanisms of antitumour activity. DNA replication in tumour cells will be blocked by the intercalation of small molecule between the base pairs of DNA [37, 38]. Generally, active compounds are required to possess approximately planar structure, with a medium-sized planar area and some hydrophobic character. In order to investigate the binding properties with DNA of Hsp and its copper(II) complexes, which have significant effect against the tested cell lines, a series of spectroscopic studies including UV-Vis, fluorescence, and CD spectra were carried out.

It is well documented that complexes copper(II) are capable of interaction with nucleic acids [39–41], and intercalative *π* ± *π* stacking of the aromatic rings of the metal complexes with the DNA bases affects the transition dipoles of the molecules and usually leads to a reduction in its absorbance [42]. The absorption titrations were carried out to determine the DNA binding of Hsp and its copper(II) complex in tris(hydroxymethyl)aminomethane (Tris) buffer. 

**Figure 3:**
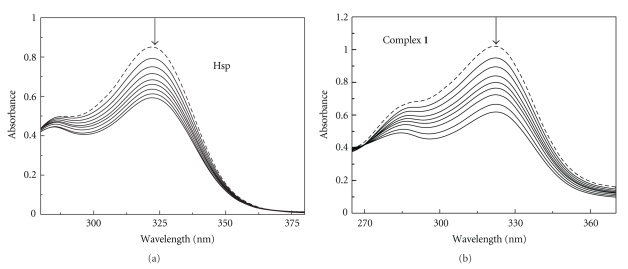
UV-Vis spectra of Hsp and complex **1** in the absence (- - - -) and presence (—) of increasing amounts DNA.

The UV-Vis absorption spectra of Hsp and complex **1** in the absence and presence of calf thymus DNA are shown in Figure 3. The results indicated that the absorption spectrum of Hsp was similar to that of complex **1** with an intense absorption band around 322 nm. After the addition of ct-DNA, the absorption bands of Hsp and complex **1** at 322 nm both exhibited obvious hypochromism about 30.3% for Hsp, 39.4% for complex **1**, although there was no obvious red shift. 

**Figure 4:**
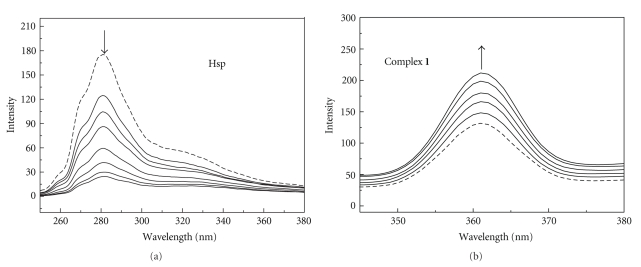
Emission spectra of Hsp and **1** in the absence (- - - -) and presence (—) of increasing amounts of DNA.

In order to investigate the interaction pattern of Hsp and complex **1** with ct-DNA, fluorescence emission titration analyses were undertaken. The fluorescence emission spectra of Hsp and complex **1 **in the absence and presence of calf thymus DNA are shown in Figure 4. Hsp showed a weak emission band around 284 nm, and complex **1** exhibited a strong emission band around 361 nm. With the addition of ct-DNA, the emission intensity of Hsp caused decrease, whereas that of complex **1 **displayed enhancement. The emission intensity decrease of Hsp in the presence of DNA may be caused by the fact that Hsp being a small hydrophobic molecule can be adsorbed by hydrophobic group on the surface of DNA. Such a characteristic change is often observed in DNA interactions [14]. The emission intensity increase of complex **1 **on addition of ct-DNA is ascribed to a decrease of the collisional frequency of the solvent molecules with the complex which caused by the planar aromatic group of the complex stacks between adjacent base pairs of ct-DNA [43]. Moreover, the emission intensity of Hsp decreased significantly reaching saturation with an decreasing ratio of 7.26 at the [DNA]/[Hsp] ratio of 8 : 1, whereas that of complex **1** enhanced reaching saturation with an incremental ratio of 1.94 at the [DNA]/[Hsp] ratio of 5 : 1. These variations may be caused by the fact that the interaction degree was related to the planar area and hydrophobic character, suggesting the binding affinity of complex **1** is stronger than that of free ligand. 

Circular dichroism (CD) is a useful technique to assess whether nucleic acids undergo conformational changes as a result of complex formation or changes in the environment [44]. The DNA helix has a right-hand chiral structure, maintaining the B conformation in solution. The CD absorption spectrum is very sensitive to conformational changes in DNA; generally, in noncovalent binding states, electrostatic and groove bindings show no significant CD spectral perturbation, because these two binding modes do not influence the secondary structure of DNA. The observed intensity change can be attributed to an intercalation mode when small planar molecules interact with DNA. 

**Figure 5:**
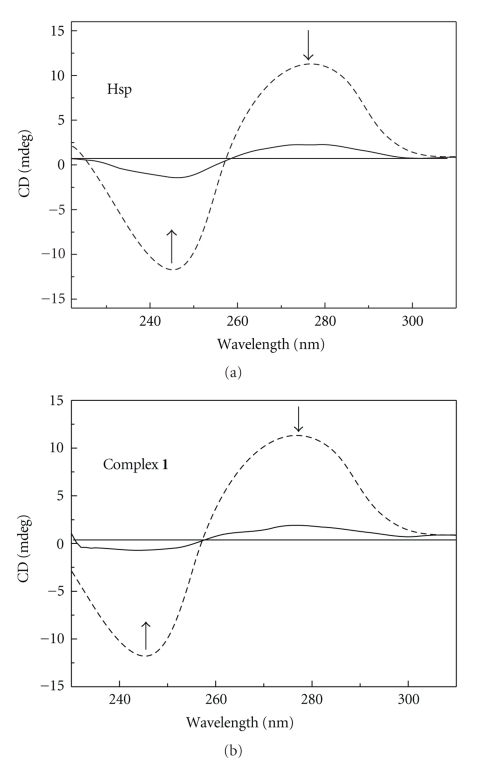
CD spectra of ct-DNA of Hsp and **1** in the absence (- - - -) and presence (—) of complexes.

 As shown in Figure 5, the CD spectra of ct-DNA show a positive band at 275 nm and a negative band at 245 nm, due to base stacking and right-hand helicity, respectively, consistent with the characteristic B conformation of DNA [45, 46]. With addition of the compounds, the intensities of the negative and positive bands decrease, but no red shift is observed. These alterations in the CD spectrum of DNA indicate strong conformational changes by Hsp and complex **1**. Also, Hsp and complex **1 **bind DNA mainly by intercalation mode. 

Based on the UV-Vis spectral, fluorescence and CD measurements revealed that, with the addition of increasing ct-DNA, complexes exhibited obviously hypochromism, fluorescence emission intensity enhancement, and CD spectrum change, which are important evidences of complex interacting with the DNA by intercalation. In summary, Hsp and complex **1 **bind DNA mainly by intercalation mode, and the binding affinity of complex **1** is stronger than that of free ligand.

In addition, interactions of complexes** 2 **and **3** and their ligands with calf thymus DNA were also investigated under the same experiment condition. Similar results were obtained, and no obvious differences in the DNA binding mode were founded.

## 4. Conclusion

Three flavonoids copper(II) complexes were synthesized, and their cytotoxic activities were estimated against HepG-2, SGC-7901 and HeLa cell lines in vitro. The results showed that complexes **1** and **3** showed higher inhibitory rate than their free ligands against SGC-7901 and HepG2 cell lines; the inhibitory rate of complex **1** was 43.2% and 43.8%, while complex **3** was 46% and 36%, respectively. In comparison with complex **2**, complexes **1 **and **3 **exhibited higher cytotoxicity towards selected cell lines, and complex **2 **showed relatively significant inhibitory rate only against HepG2 cell lines than that of Nrg. The difference in inhibition rates between the Cu(II) complexes of Apg and Nrg was presumably due to the flavone planar structure of Apg. In contrast to the inhibitory activity of the Cu(II) complexes with Nrg and Hsp, the high activity of Cu(II) complex with Hsp may be related to the different hydroxyl (OH) substitutions in ring B. The binding properties with calf thymus DNA revealed that Hsp and its Cu(II) complex bind DNA mainly by intercalation mode. It confirmed that DNA is an important target in cellular systems for these metal-based compounds derived from flavonoids. The most important contribution of this research is that the synergistic enhancement effects of flavonoids with copper(II) ion may be benefit to further development as promising potential metal-based anticancer drug.

## Figures and Tables

**Table 1:** FT-IR spectrum data (cm^−1^) for the free ligands Apg, Hsp, Nrg, and their Cu(II) complexes.

Compound	*ν* (C=O)	*ν* (C=C)	*ν* (C–OH)	*ν* (O–H)	*ν* (M–O)
Apg	1651	1608	1354	3291	—
Apg-Cu	1626	1597	1356	3413	607
Hsp	1636	1612	1360	3132	—
Hsp-Cu	1614	1564	1375	3423	598
Nrg	1629	1603	1388	3290	—
Nrg-Cu	1613	1566	1374	3401	595

**Table 2:** Thermal analytical data for complexes.

Compound	Dehydration			Decomposition		
	temperature (°C)			temperature (°C)		

	T1	T2	Weight loss (%)	T3	T4	Total weight loss (%)
Hsp-Cu	100	250	7.2	347	540	55.9
Nrg-Cu	94	117	7.9	248	440	76.5
Apg-Cu	—	247	5.2	420	580	55.9

T1–T2: temperature range corresponding to complex dehydration; T3–T4: temperature range corresponding to complex decomposition.
